# Supplementary material for: Residential Nitrogen Dioxide Exposure and Cause-Specific Cerebrovascular Mortality: An Individual-Level, Case-Crossover Study
Source: Toxics. 2023 Dec 22;12(1):10. doi: 10.3390/toxics12010010 (PMC10818787; doi:10.3390/toxics12010010)
Supplement: Supplementary file 1 [file toxics-12-00010-s001.zip › toxics-2758299-supplementary.pdf]

Supplemental Material

Contents

Figure S1. The lag structure for percent changes of cerebrovascular mortality associated with a 10 μg/m^3 increase in NO_2 over lag 0 to 14 days.

Figure S2. The cumulative exposure-response curves for the relationships between daily concentrations (μg/m^3) of NO_2 and cerebrovascular mortality in subgroups over 0 to 7 days.

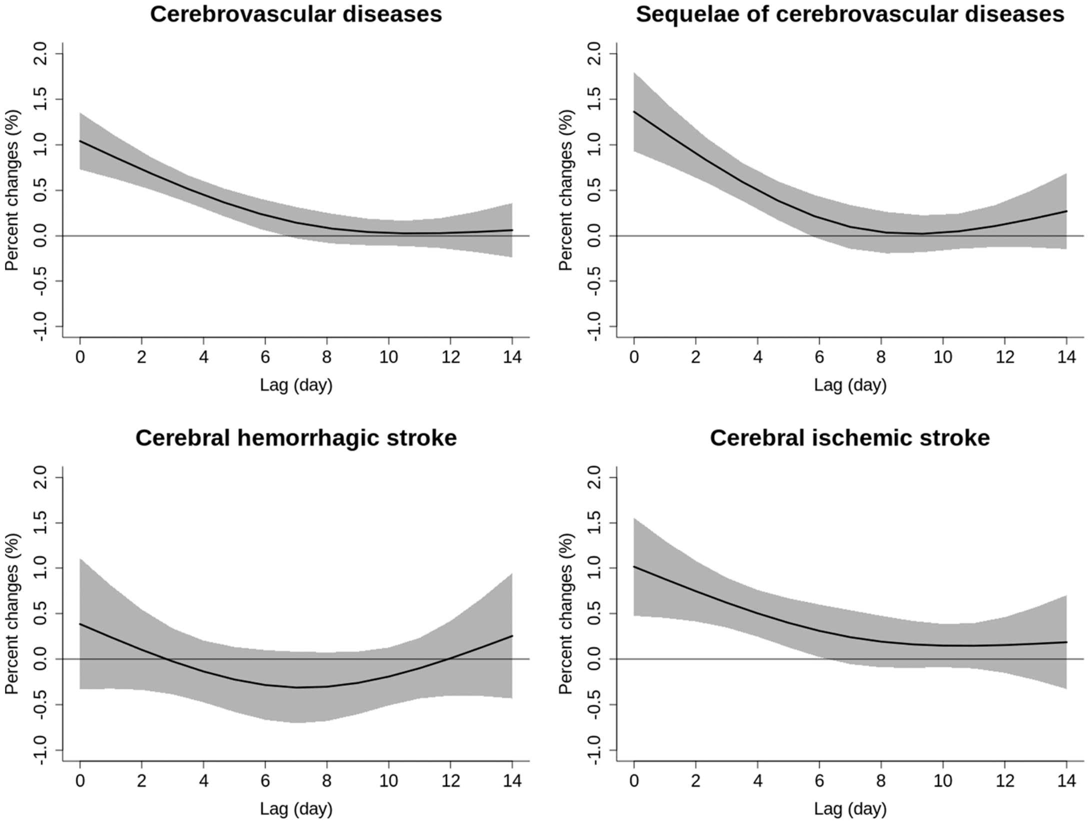

Figure S1. The lag structure for percent changes of cerebrovascular mortality associated with a 10 μg/m^3 increase in NO_2 over lag 0 to 14 days.

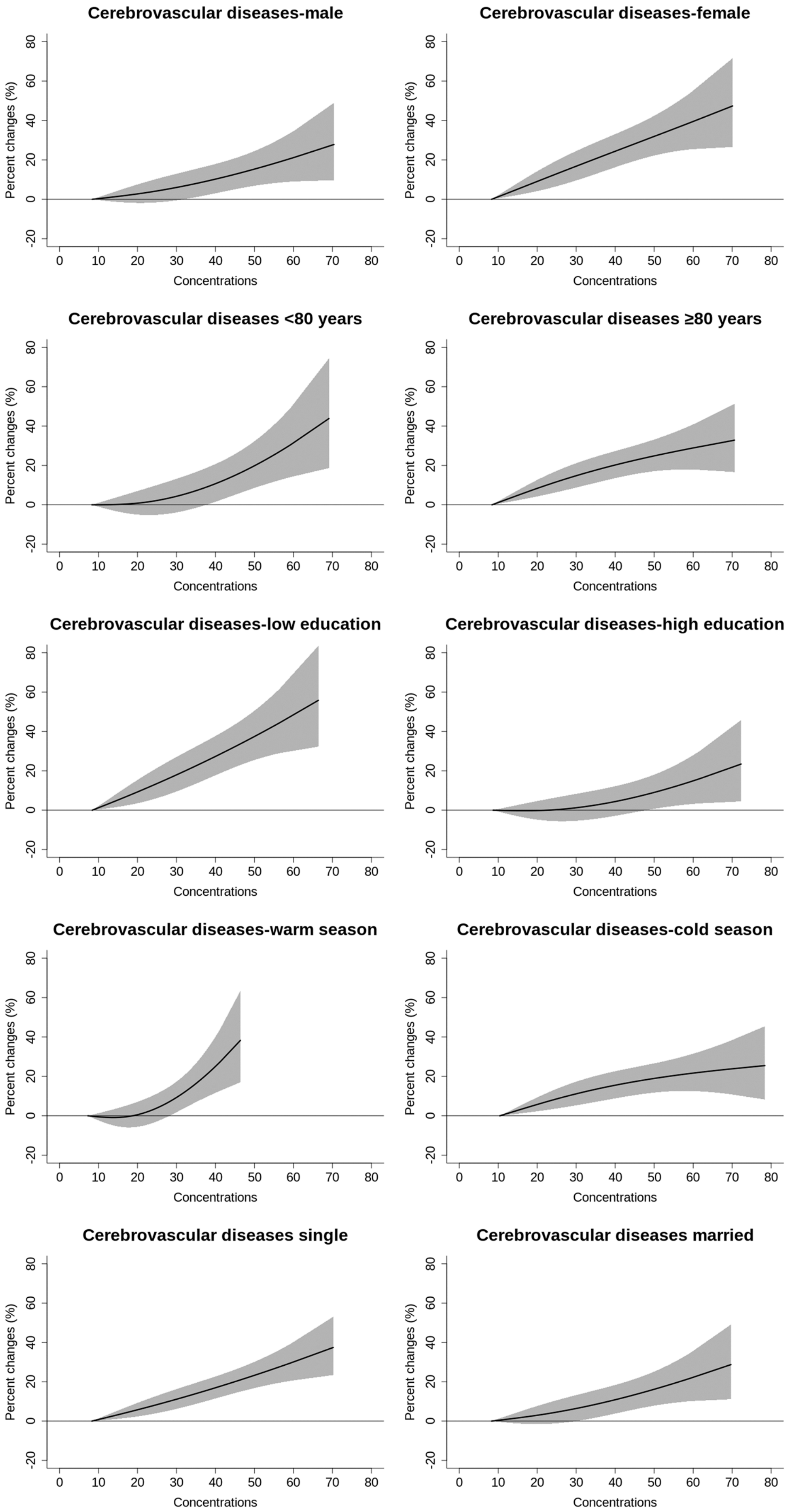

Cerebrovascular diseases-male
Cerebrovascular diseases-female
Cerebrovascular diseases <80 years
Cerebrovascular diseases ≥80 years
Cerebrovascular diseases-low education
Cerebrovascular diseases-high education
Cerebrovascular diseases-warm season
Cerebrovascular diseases-cold season
Cerebrovascular diseases single
Cerebrovascular diseases married
Percent changes (%)
-20
0
20
40
60
80
Concentrations
0
10
20
30
40
50
60
70
80

Figure S2. The cumulative exposure-response curves for the relationships between daily concentrations (μg/m^3) of NO_2 and cerebrovascular mortality in subgroups over 0 to 7 days.
